# Genetic divergence of native palms of *Oenocarpus distichus* considering biometric fruit variables

**DOI:** 10.1038/s41598-019-41507-4

**Published:** 2019-03-20

**Authors:** Gleidson Guilherme Caldas Mendes, Mônica Trindade Abreu de Gusmão, Thales Guilherme Vaz Martins, Renato Domiciano Silva Rosado, Rayanne Savina Alencar Sobrinho, Andrei Caíque Pires Nunes, Wellington Souto Ribeiro, José Cola Zanuncio

**Affiliations:** 1 Departamento de Engenharia Florestal, Universidade Federal de Viçosa, 36570-900 Viçosa, Minas Gerais Brazil; 2 Instituto de Ciências Agrárias, Universidade Federal Rural da Amazônia, 66077-030 Belém, Pará Brazil; 3 Departamento de Estatística Aplicada e Biometria, Universidade Federal de Viçosa, 36570-900 Viçosa, Minas Gerais Brazil; 4 Centro de Formação em Ciências Agroflorestais, Universidade Federal do Sul da Bahia, 45613-204 Itabuna, Bahia Brazil; 5 Programa de Pós-graduação em Horticultura Tropical, Universidade Federal de Campina Grande, 8, Rua Jairo Vieira Feitosa, 58840-000 Pombal, Paraíba Brazil; 6 Departamento de Entomologia/BIOAGRO, Universidade Federal de Viçosa, 36570-900 Viçosa, Minas Gerais Brazil

## Abstract

*Oenocarpus distichus* presents economic, ecological, and dietary potential for pulp market processed *in natura*. Germplasm conservation and genetic improvement depend on genetic divergence studies. The objective was to quantify genetic divergence in a native population of *O. distichus* genotypes based on fruit biometrics. The fruit length and width, fruit mass, pulp and seed, pulp and almond thickness, and pulp yield per fruit were evaluated. All fruit biometric characteristics of *O*. *distichus* palms show genetic variability. Genetic variations among genotypes are essential for predicting heredity and heterosis, which are essential for improving *O*. *distichus* production. Pulp yield and seed mass were negatively correlated. Almond thickness and pulp, seed mass and transverse diameter were positively correlated. Genetic distances between pair of genotypes ranged from 0.07 to 48.10 with three genetically distinct groups. The seed mass, almond thickness and transverse diameter contributed to genetic divergence. Heritability estimates the genetic control that can be obtained from *O*. *distichus* germplasm. Correlations between the variable pair reduce the evaluation effort and the resources to measure the genotype allocations in heterogeneous groups presenting high genetic variability. This makes it possible to select individuals for hybridization programs with F1 generation gains. Correlation and relative contribution networks, based on relationships graphical between fruit biometric characteristics, allow the variables selection with less effort and fewer measurements. *O*. *distichus* fruit biometric characters are efficient to quantify genetic divergence between genotypes.

## Introduction

The genetic diversity of Brazilian Amazonian plants is significant but remains poorly understood. The sites identification for conservation and germplasm collection in this region is a great challenge, especially for some native palm trees^1^.

Palm fruits and palm hearts native to the Amazon are consumed by local communities but are poorly known at the national and international levels^2^. *Oenocarpus* spp. plants have economic, ecological and dietary potential and are exploited through extraction by Amazonian communities^1,3^.

Six species of the genus *Oenocarpus* spp. are native to Brazil, but not endemic, including *O*. *distichus* Mart., *O*. *bacaba* Mart., *O*. *minor* Mart. and *O*. *mapora* H. Karten and *O*. *pataua* Mart.^4^. *Oenocarpus distichus* (also known as bacaba-de-leque) has high potential in the palm and processed pulp markets, including its use in the production of “bacaba wine”, a nutritious energy drink with commercial potential similar to that of *Euterpe oleracea* Mart.^5^.

*Oenocarpus distichus* phytochemicals have phenolic compounds correlated with antioxidant capacity^2,6,7^ and vitamin E, riboflavin, insoluble fiber, energy, and minerals, especially potassium and calcium^8^. This plant has oil with organoleptic properties similar to those of olive oil and raw material for the food industry with proteins of high biological value (40% more than soy)^5^ and adequate to manufacture ice cream and soap^9^.

The genetic diversity in *O*. *distichus* native populations, from the morphological characteristics of their fruits, needs to be better understood for germplasm inclusion. This is necessary for breeding programs to conserve native populations in extractive areas. *Oenocarpus distichus* genetic improvement and conservation programs depend on fruits biometric characterization and genetic parameter studies of genotypes showing promise for germplasm banks and conservation programs of this plant^4,10^.

Multivariate analyses allow selecting individuals for breeding programs and morphological or molecular characteristic identification to evaluate the divergence of native and exotic plants such as *E*. *oleracea*^11^, *Theobroma grandiflorum* Willd. Ex Spreng.^12^, *Elaeis guineenses* Jacq.^13^ and *Phoenix dactylifera* L.^14^ in forest fragments^15^. Grouping methods, based on prefixed similarity/dissimilarity measures, can identify divergent groups. Group determination, based on a standard, universally, accepted method does not exist, but individuals of the same group should be as homogeneous as possible and different from the others^16^.

Fruit characteristics are important for characterization, individual selection and genotypes choice during recombination cycles^15,17^. Genetic divergence, important for prospecting in plant breeding programs, allows us to know the available germplasm to evaluate similar or divergent groups to identify hybrid combinations with greater heterozygosity and heterotrophic effect^18^. Genetic divergence will allow us to know the available germplasm to predict combinations with higher heterozygosity so that there will be more possibilities of recovering fruitful *Oenocarpus distichus* genotypes in the segregant generations. Distribution among groups will allows the early determination of the best combinations and reduces the unneeded crosses number, prioritizing more contrasting groups for obtention of combination with higher heterosis. Biometric fruit variables differences such as morphological and production are regarded in the dissimilarity quantification. Those genetic variations among *Oenocarpus distichus* genotypes are essential for predicting heredity and level of heterosis, which are essential for improving *Oenocarpus distichus* production.

The objective of the present study was to quantify the genetic divergence between *Oenocarpus distichus* genotypes in a native population in the northeastern region of Pará, Amazon rainforest, Brazil, based on fruit biometric characteristics.

## Results

All *O*. *distichus* fruit biometric characteristics (F-test < 0.01) show genetic variability (Table 1) with residual variation coefficients (CV_e_) of 6.67% for fruit length (FL) at 15.09% for pulp mass (PM), indicating high experimental precision. The genetic variation coefficient (CVg) values, mainly of the PM, seed mass (SM) and fruit mass (FM) characters, confirmed the genetic variation between the *O*. *distichus* genotypes. The relationship between CV_g_/CV_e_ was greater than 1 for the characteristics fruit width (WF), FM, PM and SM. Matrix inheritance estimates for average ones (h^2^_mp_) ranged from 85.38 for pulp thickness (PT) to 96.81 for FM. The mean values of biometric characteristics of *O*. *distichus* fruits and Scott-Knott test at 5% probability are given in supplementary information.

**Table 1:** Variance analysis and genetic parameters estimates for morphological characters of *Oenocarpus distichus* fruits.

Characters	GMS	RMS	Mean	CV_e_ (%)	CV_g%_	CV_g_/CV_e_	h²_mp_
FL (mm)	13.45**	1.20	16.40	6.67	5.70	0.85	91.09
WF (mm)	15.16**	0.99	14.76	6.76	6.82	1.01	93.45
FM (g)	2.60**	0.08	2.54	11.16	16.72	1.50	96.82
PM (g)	0.40**	0.02	0.86	15.09	19.27	1.28	95.81
SM (g)	1.30**	0.06	1.68	14.23	17.75	1.25	95.61
PT (mm)	0.25**	0.04	1.65	11.56	7.47	0.65	85.38
AT (mm)	3.06**	0.19	5.53	7.86	8.19	0.99	93.82
PY (%)	164.91**	18.99	34.06	12.80	9.48	0.74	88.48

FL- fruit length; WF- fruit width, FM- fruit mass; PM- pulp mass; SM- seed mass; PT- pulp thickness; AT- almond thickness; PY- pulp yield per fruit; GMS- genotype mean square; RMS- residue mean square; CV_e_- experimental variation coefficient; CV_g_- genetic variation coefficient; h²_mp_- mean progeny heritability level; **Significant at 1% by F test.

The biometry correlation matrix of the O. distichus fruits indicate estimates for the last two eigenvalues near to zero, resulting in two linear relationships determining the harmful effects of multicollinearity (Table 2). The condition number for this matrix indicated NC > 1000, that is, severe colinearity. The eigenvectors, associated with the last two eigenvalues destacados, indicated that the FM and PM characteristics (highest absolute values highlighted in bold) are responsible for multicollinearity. The new NC matrix lower than 100, after the exclusion of the FM and PM variables, indicates weak collinearity (Table 3).

**Table 2:** Estimates of eigenvalues and eigenvectors obtained from the correlation matrix between the biometric and eigenvector evaluations associated with eight fruits morphological characters of *Oenocarpus distichus*.

Eigenvalue	Elements of the eigenvectors associated with the variables							
	FL	WF	FM	PM	SM	PT	AT	PY
5.483	0.34	0.37	0.41	0.38	0.38	0.38	0.40	0.03
1.606	−0.17	0.27	−0.15	0.30	−0.37	0.19	−0.13	0.77
0.400	0.79	−0.22	0.06	0.10	0.03	−0.34	−0.36	0.26
0.314	0.46	0.15	−0.40	−0.44	−0.32	0.53	0.10	−0.12
0.136	0.04	0.84	−0.06	−0.09	−0.04	−0.43	−0.21	−0.21
0.058	0.12	−0.04	−0.17	−0.02	−0.23	−0.49	0.80	0.16
0.003	0.08	−0.08	−0.10	**0**.**68**	−0.52	0.03	−0.05	−0.50
0.000	0.00	0.00	−**0**.**78**	0.31	0.55	−0.00	0.00	−0.00

FL- fruit length; WF- fruit width, FM- fruit mass; PM- pulp mass; SM- seed mass; PT- pulp thickness; AT- almond thickness and PY- pulp yield per fruit. Highest absolute values highlighted in bold in Table 2 indicate the eigenvectors, associated with the last two eigenvalues.

**Table 3:** Eigenvalues and eigenvectors associated with six morphological characters of *Oenocarpus distichus* fruits.

Eigenvalue	FL	WF	SM	PT	AT	PY
3.852	0.42	0.44	0.44	0.45	0.48	−0.00
1.420	−0.14	0.33	−0.37	0.26	−0.09	0.81
0.397	0.85	−0.17	−0.00	−0.25	−0.34	0.26
0.160	0.18	−0.50	−0.45	0.69	0.06	−0.18
0.119	0.16	0.65	−0.47	0.02	−0.32	−0.49
0.053	0.15	−0.00	−0.50	−0.43	0.74	0.02

FL- fruit length; WF- fruit width, SM- seed mass; PT- pulp thickness; AT- almond thickness and PY- pulp yield per fruit.

In addition to the CN, small single values were observed for FM (0.0503) and PM (0.0043) and high condition index FM (46.60) and PM (537.90), indicating serious problems caused by multicollinearity. VIF values can also be used to detect the existence of multicollinearity. The FM and PM variables presented VIFs of 12.53 and 101.37, respectively. The existence of at least one VIF with a value greater than 10 is sufficient for the regression coefficients associated with these values to be highly influenced by multicollinearity, indicating that these variables must be removed from the analyzes^19^.

**Figure 1:**
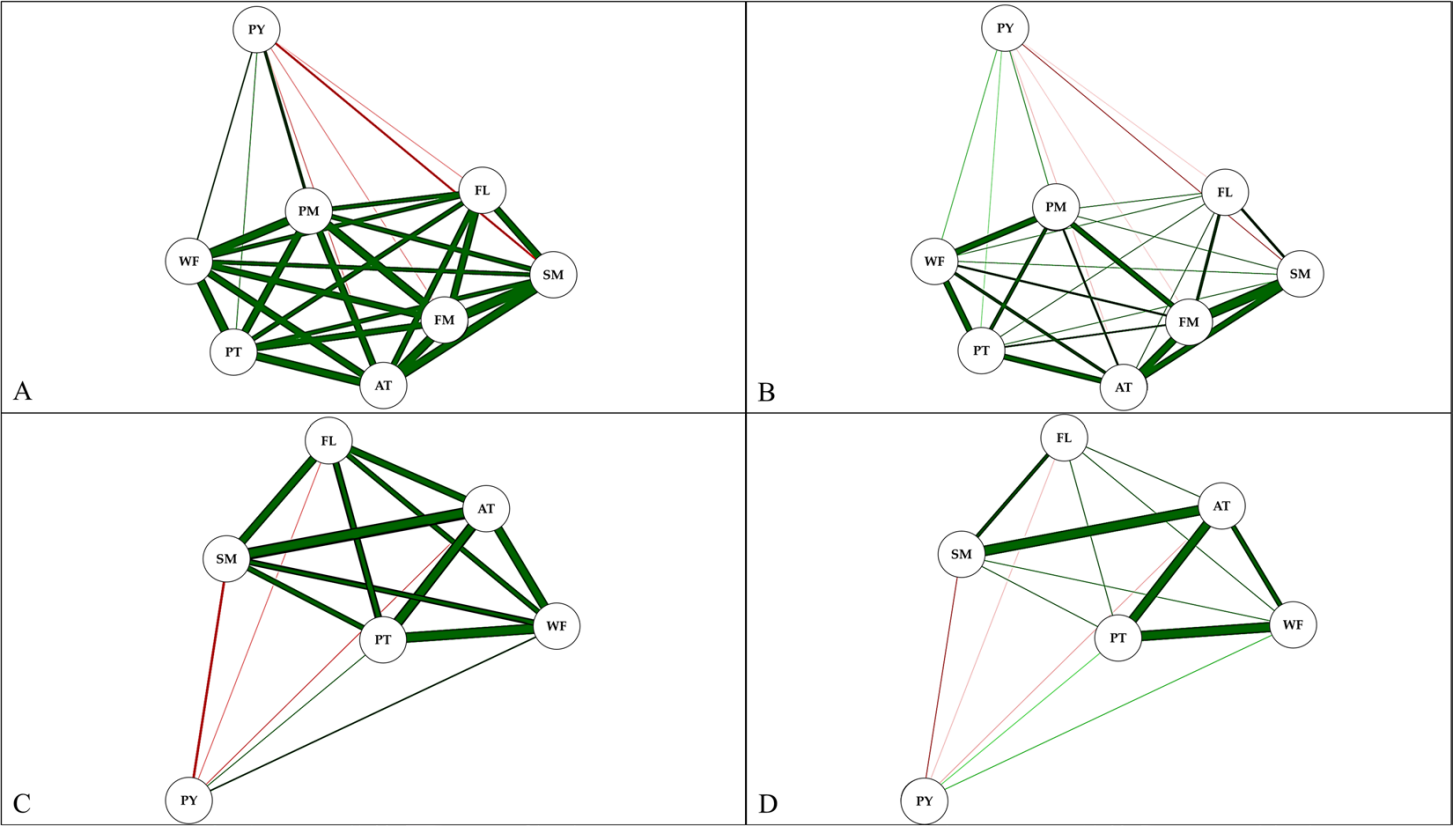
Phenotypic correlation network of biometric characters fruit length (FL); fruit width (WF); fruit mass (FM); pulp mass (PM); seed mass (SM); pulp thickness (PT); almond thickness (AT) and pulp yield per fruit (PY) considering: (**A**,**B**) correlation network under the multicollinearity effect (NC > 1000) and (**C**,**D**) without multicollinearity effect (NC < 100), (**A**,**C**) correlation > 0.3 and (**B**,**D**) correlation > 0.7. Green lines mean positive correlation and negative redlines. The line thickness is proportional to the correlation intensity. Phenotypic correlation network generated with GENES SOFTWARE (Genes Software – extended and integrated with the R, Matlab and Selegen. http://arquivo.ufv.br/dbg/genes/genes_Br.htm).

The PM characteristic correlated with FL and PT and FM with AT and SM, all above 70% (Fig. 1B). The correlation pattern between the fruit biometric characteristics did not vary, even after excluding the FM and PM variables that caused multicollinearity (Fig. 1C,D). The characteristics PY and SM (Fig. 1C) and AT, SM, PT and WF (Fig. 1D) correlated, negatively, with a magnitude of 42% and above 70%, respectively.

**Figure 2:**
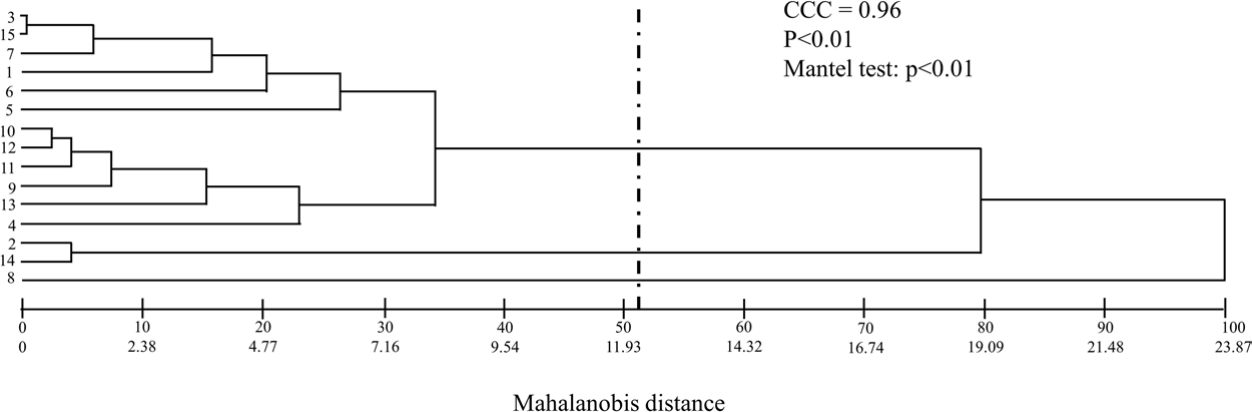
Dendrogram obtained by unweighted pair group method with arithmetic mean (UPGMA) based on Mahalanobis distance of fifteen *Oenocarpus distichus* genotypes with the cut-off point determined according to^20^ and cophenetic correlation coefficient (CCC). Dendrogram generated with GENES (GENES - a software package for analysis in experimental statistics and quantitative genetics. http://arquivo.ufv.br/dbg/genes/genes_Br.htm).

Genetic distances between genotype pairs of *O*. *distichus* ranged from 0.07 to 48.10, with a mean of 11.44 (Table 4). Genotypes 3 and 15 formed the least divergent pair and 2 and 8 formed the most divergent one. The mean distances for 39% of genotype pairs were above the overall mean. The grouping of Tocher separated *O*. *distichus* genotypes into three genetically distinct groups (Table 5) with 80% of the genotypes in group I and a single palm in III (8), being the most divergent. The UPGMA method showed several groups, but a cut based^20^ reduced them to only three divergent genotype groups. The stratification in genetically different groups was equal to that of Tocher, with most of the genotypes in group I, and group III presenting only one genotypes at 8 (Fig. 2). The calculated cophenetic correlation coefficient (r = 0.96) in this study indicated that how similar the final hierarchical pattern and initial similarity (or distance) matrix are. Moreover, were obtained distortion and stress of 16 and 20%, respectively.

**Table 4:** Matrix (M) of genetic dissimilarity between *Oenocarpus distichus* genotypes based on the generalized distance of Mahalanobis (D²).

M	2	3	4	5	6	7	8	9	10	11	12	13	14	15
1	21.48	3.61	12.06	7.27	5.47	3.85	16.34	8.86	6.61	3.53	5.54	7.16	19.31	3.83
2		21.76	21.10	43.82	18.97	29.37	48.10	9.93	10.51	13.02	13.36	14.53	0.95	21.71
3			7.86	5.01	4.52	1.39	19.74	4.05	4.00	2.24	3.80	7.92	20.33	0.07
4				16.33	9.03	11.04	13.32	7.19	5.93	5.10	3.51	5.72	18.35	7.17
5					9.78	3.87	14.84	17.34	16.77	11.73	14.74	19.88	42.64	5.62
6						4.42	19.46	8.30	7.42	3.78	6.47	13.13	18.83	4.94
7							20.25	8.76	7.61	3.75	6.53	11.76	26.93	1.43
8								30.12	26.77	20.40	20.73	19.16	45.14	19.84
9									0.64	2.61	2.05	5.52	8.98	3.75
10										1.23	0.59	3.29	8.41	3.52
11											0.70	3.68	11.50	1.90
12												2.16	10.49	3.21
13													10.40	7.09
14														19.85

**Table 5:** Dissimilarity groups formed by the Tocher method from the generalized distances of Mahalanobis estimated for *Oenocarpus distichus* genotypes.

Groups	Palms
I	3 15 7 1 6 5 10 12 11 9 13 4
II	2 14
III	8

**Figure 3:**
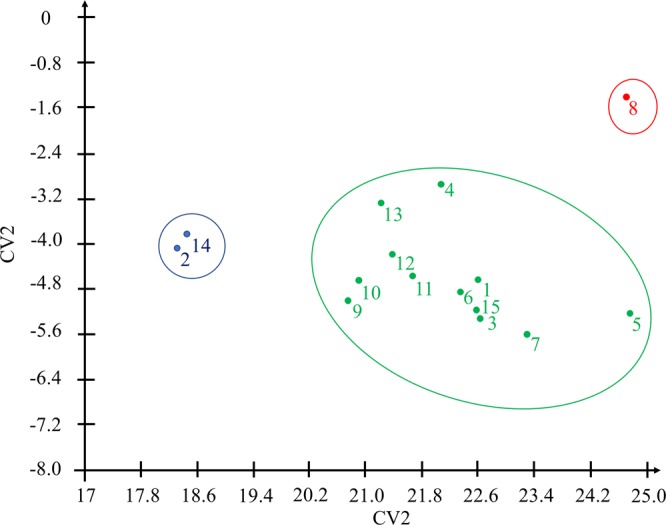
Canonical variable 2D biplot related to six fruits traits of fifteen *Oenocarpus distichus* genotypes. Cophenetic correlation coefficient, distortion and stress of 96%, 16% and 20%, respectively. 2D biplot generated with GENES (GENES - a software package for analysis in experimental statistics and quantitative genetics. http://arquivo.ufv.br/dbg/genes/genes_Br.htm).

The first two canonical variables explained more than 80% of the total data variation (Table 6). The relative contribution of the fruit biometric variables to the genetic divergence was from 9.00 to 39.88% among the *O*. *distichus* palms and SM, AT and WF, with the greatest contribution to genetic divergence. The graphic dispersion in the two-dimensional space of 15 genotypes of *O*. *distichus* in relation to the first two canonical variables (Fig. 3), after exclusion of the least important variable, FL (CR = 9%), allowed the grouping of accessions similar to the optimization methods (Table 6) and UPGMA (Fig. 2).

**Table 6:** Relative contribution of variables (CR), eigenvalue estimates (AV), accumulated variance percentages (AC%) and weighting coefficients (eigenvectors) associated with each variable by canonical ones.

VC	Variance		Matrix of eigenvectors associated with variables					
	AV	% AC	FL	WF	SM	PT	AT	PY
1	3.41	59.52	−0.02	0.11	4.07	2.55	0.80	0.15
2	1.20	80.44	0.61	−1.00	1.43	−0.29	0.22	−0.08
3	0.54	89.95	−0.45	0.20	−2.87	2.11	1.63	−0.23
4	0.37	96.33	0.82	0.00	−4.05	2.13	0.55	−0.11
5	0.17	99.28	0.06	0.78	0.29	−0.60	−1.62	−0.13
6	0.04	100.00	0.11	0.12	−0.90	−**3**.**84**	1.13	0.01
	CR (%)		9.00	13.71	39.87	11.66	15.20	10.54

FL- fruit length; WF- fruit width, SM- seed mass; PT- pulp thickness; AT- almond thickness and PY- pulp yield per fruit.

## Discussion

The genetic variability between the *O*. *distichus* genotypes, for all fruit biometric characteristics, indicates that the germplasm of this plant can be collected in the region studied. Additionally, the selection, based on any of the eight features, provides genetic advantages to differentiate individuals within the same population, due to the cross between genetically different individuals causing greater heterotrophic effect^21^. The variability between the genotypes can be attributed to the data quantitative nature, polygenic, controlled by many genes^13,22^ and is important in *ex situ* genetic conservation, due to increasing the effective population size and reducing the mortality caused by inbreeding^23^. The efficiency of these biometric characteristics to distinguish genotypes from the family Arecaceae was confirmed with similar results for *E*. *oleracea*^11–21^. The CV_g_/CV_e_ ratio (>1) and the high h²_mp_ values, confirm the variability and the genetic control of fruit characteristics, allowing selecting and recombining the most promising *O*. *distichus* genotypes. CV_g_/CV_e_ values, greater than 1, indicate gains in selection^24^ and that the environment has a low impact on the characteristics measured. These values should be considered in breeding and conservation programs for this palm^10^. Genetic variations in native populations are important to obtain parents to produce superior and divergent individuals for the interest characteristics in the F1 generation^22,25,26^. However, collection intensity may reduce fruit availability for frugivorous animals, compromising gene flow and genetic diversity in the forest fragment^27^. Therefore, conservation strategies such as not harvesting the fruits in years of low productivity and maintenance of at least 50% of them on the palms are important to maintain consumption by wildlife^28^.

The high multicollinearity values caused by FM and PM were expected, because they are characteristics collinear with PY and common in studies involving fruit characteristics, as reported for *Theobroma grandiflorum* Willd. Ex Spreng.^12^ and *Plukenetia volubilis* L.^29^. However, they should be excluded from the analyses, because they may lead to false biological interpretations and errors in the genotype selection process, due to the lack of precision in parameter estimation.

The correlations between the fruit biometric variables, visualized by graphic network analysis, show the potential of this technique to select variables that reduce the evaluation time and resources for measurement in *O*. *distichus* breeding programs. This confirms their wide use to relate characteristics of interest and to enable greater effectiveness in the divergent selection and superior *O*. *distichus* genotypes, as reported for *Capsicum* spp. Species^30^, *Passiflora edulis* Sims^31^ and *Cocos nucifera* L.^22^.

The large number of genotypes with genetic distances above the general average confirms genetic divergence and suggests the crosses’ ability to explore additive genetic values in *O*. *distichus* individuals and consequently their heterosis in subsequent cycles, as reported for *P*. *volubilis*^29,32^. Similar groupings with the Tocher and UPGMA methods, the consistent genetic divergences between genotypes 2, 5 and 8 and the low number of groups may indicate a common ancestry for this plant in the region^12^, similar to that reported for *Bactris gasipaes* Kunth.^33^, *E*. *oleracea* Mart.^11^ and *T*. *grandiflorum* Willd. Ex Spreng.^12^ with 3, 5 and 3 groups, respectively. Matrix separation into heterogeneous groups may indicate distinct gene pools for controlled hybridizations and reciprocal recurrent selection and, therefore, be interesting for genetic improvement^18^. Additionally, the grouping restricts the crosses number, reducing costs and increasing genetic gains in subsequent cycles, due to the reduced in breeding coefficient in the population by excluding similar individuals^32^.

The concentration of a large part of the total variance in the first two canonical variables (above 80%) shows that it is possible to study the genetic divergence between *O*. *distichus* genotypes by geometric distances in scatter plots^34^, as reported for genetic divergence in *C*. *nucifera*^22^. The projection of dissimilarity measures in 2D projection representation and use of dendrograms are acceptable when the correlation value between measures of original distances and a graphics is higher than 0.9 and values of distortion and stress lower than 20%^35^. In a graphic dispersion of canonical variables the grouping was compatible with the ones grouped by Tocher’s method. The bigger contribution of the SM, AT and WF characteristics to genetic divergence is important in selection programs, for being more responsive in the superior genotype identification^32^. The variable FL, even presenting lower relative contribution to genotype divergence, should not be removed from the analyses because it is easy to determine using non-destructive methods^36^. In addition, most of the genotypes that had lower FL showed higher yields of pulp (supplementary information). This result is in agreement with what is reported in the literature for *E*. *oleracea*, where higher fruit lengths result in lower pulp yields^37^.

## Conclusions

Biometric fruit characteristics are efficient to quantify genetic divergence between *Oenocarpus distichus* genotypes. The contribution of the seed mass, almond thickness and fruit width, for genetic divergence, is higher and for this reason, these parameters should be prioritized in selection processes. Correlation networks facilitate the visualization of the correlations between the variables and the potential to choose those requiring less time for evaluation and fewer resources in the measurements in *O*. *distichus* breeding programs.

Grouping methods are effective in allocating *O*. *distichus* genotypes, in different groups, and should be used to maintain the genetic variability, conservation and selection of genotypes of this plant for breeding programs in native populations.

## Material and Methods

### Study area characterization

**Figure 4:**
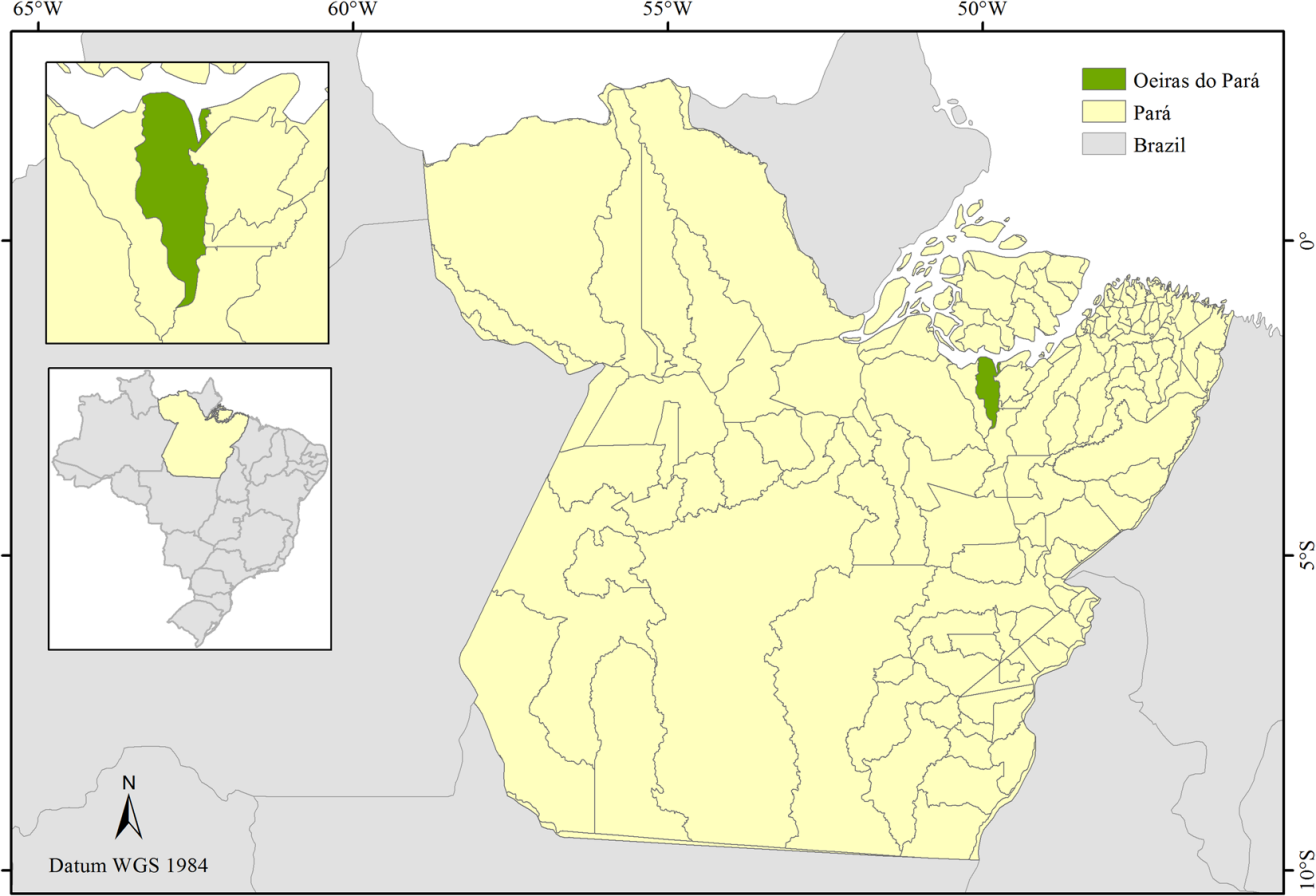
Geographic location of the municipality of Oeiras do Pará, Pará, Brazil. Map generated with ArcGis 10.6.1 (ESRI Development Team, 2018. ArcGis Geographic information system (GIS) for working with maps and geographic information. https://www.esri.com).

Fruits were collected from *O*. *distichus* native genotypes in an Amazonian forest fragment in the municipality of Oeiras do Pará, northeast of Pará, Brazil (02°00′11″S; 49°51′16″W) (Fig. 4). The climate is Ami type, according to Köppen’s classification, with 2.334 mm year^−1^ annual precipitation concentrated from December to March. The annual average temperature and relative air humidity is 83% and 29 °C, respectively^38^. The original forest types are dense low plateau forests and dense alluvial, with high populations of the genus *Oenocarpus*.

### Plant material

*Oenocarpus distichus* genotypes, with mature fruits, were selected and those in good phytosanitary conditions were collected in April 2016, based on fruit productivity and with a minimum distance of 150 meters between genotypes. The fruits of each genotypes were identified, packed in plastic bags, placed in a Styrofoam box with ice and transported to the genetics and improvement laboratory of the Universidade Federal Rural da Amazônia, Campus Belém, Pará.

### Biometric and statistical analysis

**Figure 5:**
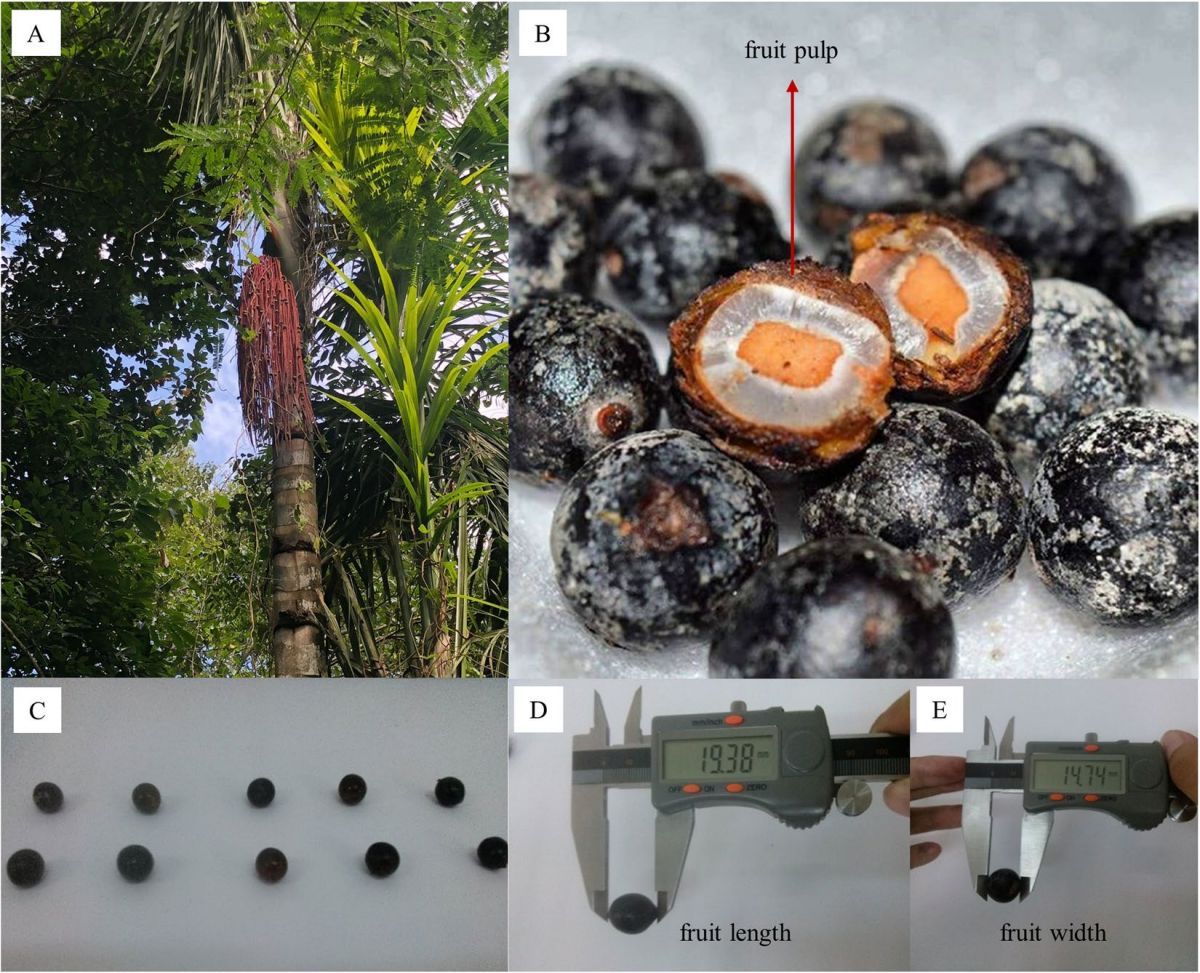
*Oenocarpus distichus* palm (**A**), fruit pulp (**B**), fruits of different sizes (**C**), fruit length (**D**) and fruit width (**E**). Photos were taken by Gleidson Guilherme Caldas Mendes.

The statistical design was entirely random, with 15 genotypes and 14 fruits collected from each one. The length (FL), width (WF) (mm) and mass (FM) of fruits, pulp (PM) and seed (SM) (g) mass; and pulp (PT) and almond (AT) (mm) thickness were evaluated (Fig. 5). The pulp yield per fruit (PY) was obtained by the ratio between the FM and PM. The masses were determined on a digital scale (0.001 g) and the length and width using pachymeter (0.01 mm).

Genetic variability between genotypes was tested with analysis of variance (ANOVA using a Fisher’s test (F-test) at 1% probability. The genetic parameters heritability (h^2^_mp_), coefficient of genetic variation (CV_g_%) and variation coefficient (CV_e_%) were also determined. The means were grouped by Scott-Knott method^39^.

Possible linear relationships in the residual correlation matrices were obtained with multicollinearity test^40^. The multivariate analysis of *O*. *distichus* fruit data was performed using canonical variable techniques^41^. Dissimilarity was determined with the Mahalanobis generalized distance^42^ with grouping technique. The Tocher optimization method^41^ with the divergence measure mean per group lower than those between any other, and using the arithmetic mean method between unweighted pairs (UPGMA-Unweighted Pair Group Method with Arithmetic Average)^43^ based on Mojena^20^ delimited the groups. The relative contribution of the characteristics to genetic divergence was quantified by Mahalanobis generalized distances using the Singh method^44^.

Relationships between the fruit biometric evaluations were represented graphically with correlation networks. The phenotypic correlation were analyzed by weighted matrix with connections between the variables determined by the adjacent matrix *A* = *h (R)* as a function of: *H*(*r*_*ij*_) = ½ {*sgn* (*│r*_*ij*_*│−ρ*) + 1}, in which *ρ* (hard limit) = 0, which allows viewing the connections. Network graphs were obtained with the variables with positive correlations connected by a green line and the negative ones by a red line. The line’s thickness represents the absolute correlation value with the thicker the line. The line’s thickness was controlled with a cut-off value of 0.3 and 0.7, meaning that only |rij| ≥ 0.3 and |rij| ≥ 0.7 have their lines highlighted, for easy viewing. The layout for the network was created with Fruchterman-Reingold algorithm^45^. The analyses were performed with Genes software^46^ and integration was realized with R^47^. The integration of the correlation network was evaluated with the “Qgraph” package^48^.
